# Early-life and pubertal stress differentially modulate grey matter development in human adolescents

**DOI:** 10.1038/s41598-018-27439-5

**Published:** 2018-06-15

**Authors:** Anna Tyborowska, Inge Volman, Hannah C. M. Niermann, J. Loes Pouwels, Sanny Smeekens, Antonius H. N. Cillessen, Ivan Toni, Karin Roelofs

**Affiliations:** 1 Behavioural Science Institute, Radboud University Nijmegen, Montessorilaan 3, 6525 HR Nijmegen, The Netherlands; 2 Donders Institute for Brain, Cognition and Behaviour, Radboud University Nijmegen, Kapittelweg 29, 6525 EN Nijmegen, The Netherlands; 3 Sobell Department of Motor Neuroscience and Movement Disorders, UCL Institute of Neurology, University College London, Queen Square, London, WC1E 6BT United Kingdom; 4 Faculty of Psychology and Educational Sciences, Open University of the Netherlands, Valkenburgerweg 177, 6419 AT Heerlen, The Netherlands

## Abstract

Animal and human studies have shown that both early-life traumatic events and ongoing stress episodes affect neurodevelopment, however, it remains unclear whether and how they modulate normative adolescent neuro-maturational trajectories. We characterized effects of early-life (age 0–5) and ongoing stressors (age 14–17) on longitudinal changes (age 14 to17) in grey matter volume (GMV) of healthy adolescents (n = 37). Timing and stressor type were related to differential GMV changes. More personal early-life stressful events were associated with larger developmental reductions in GMV over anterior prefrontal cortex, amygdala and other subcortical regions; whereas ongoing stress from the adolescents’ social environment was related to smaller reductions over the orbitofrontal and anterior cingulate cortex. These findings suggest that early-life stress accelerates pubertal development, whereas an adverse adolescent social environment disturbs brain maturation with potential mental health implications: delayed anterior cingulate maturation was associated with more antisocial traits – a juvenile precursor of psychopathy.

## Introduction

Adolescence is a critical developmental stage during which a cascade of biological changes leads to profound structural modifications in the brain. The protracted maturation of human adolescents^1,2^ makes brain development particularly sensitive to ongoing environmental stressors^3^. Studies in clinical populations and animal models have also shown that neurodevelopmental trajectories are influenced by incubated effects of early-life stressors^4,5^. In this study we examined the effects of early-life stress, experienced from birth until 5 years of age, on brain developmental trajectories of healthy adolescents (14–17 years old), while also considering cerebral effects of ongoing stressors.

Cross-sectional studies have provided converging evidence for the effects of early-life and pubertal stress on developmental susceptibility of grey matter volume (GMV)^6,7^. However, inferences on developmental trajectories require longitudinal designs. Previous neurodevelopmental studies on the effects of early-life adversity have used longitudinal designs in (sub-) clinical cohorts^8–10^, but those studies cannot distinguish general developmental effects from cohort-specific effects. By discriminating between the effects of different types of stress, exerted at different developmental times, we characterize the stress susceptibility of grey matter maturation in a normative sample during the final window of pubertal plasticity^11–13^.

Stress activates the production of glucocorticoids that influence receptors distributed throughout the brain, with particularly high concentrations in the prefrontal cortex, hippocampus, and amygdala^14,15^. These stress-sensitive brain regions are particularly susceptible during adolescence, when hormonal stress sensitivity is enhanced^16,17^, leading to stress-related reductions in GMV^18–21^. One interpretation of volumetric reductions, particularly in animals and adults, has been linked to the toxic effects of glucocorticoids causing dendritic spine loss or even cell death^3,22^. However, recent studies have interpreted structural changes, particularly during adolescence, as accelerated maturation of neural circuits associated with emotional processing due to an evolutionary prioritization of adult-like functioning^23^. Brain volumetric changes may be the net result of differential effects of current stress and stress experienced early in life, different maturational profiles of prefrontal and limbic structures, and the interaction between stress occurrence and developmental state^1,24–26^. Beside timing of stress occurrence, the nature of the stressors might also diversely impact neurodevelopmental trajectories^27^. Here we consider two distinct stressor categories, that is personal negative life events (such as illness, parental divorce, etc.) and adverse social environments. For the latter, we account for the fact that children of different ages are predominantly sensitive to different social environments – the relationship with parents has a profound impact during early childhood^28,29^, whereas peer relationships become increasingly important during adolescence with poor relations forming a potent stress-factor in that time-window^30,31^.

**Figure 1:**
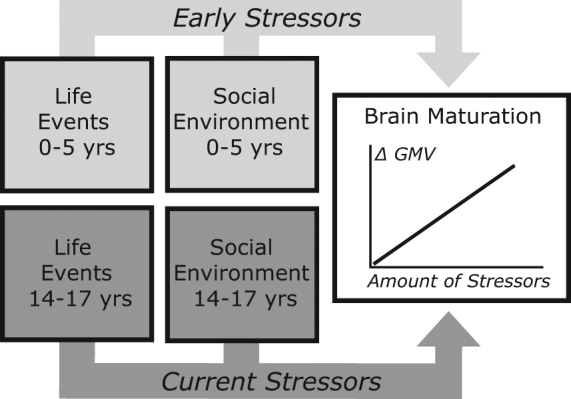
Model of early childhood and current adolescent factors influencing pubertal neural development. The amount of stressors, i.e. negative personal life events and social environment, affect the magnitude of change (positive or negative) in grey matter volume (GMV).

This study disambiguates cerebral effects of early childhood events from current pubertal stress, evoked by personal and by social circumstances, on neurodevelopmental trajectories. Those trajectories are estimated from a structural index of brain development (GMV) measured between mid and late adolescence in 37 adolescents tested at 14 and 17 years of age (Fig. 1). We apply whole-brain statistical inferences. We expect that regions with a high distribution of glucocorticoid receptors will show stress-effects on brain development^32^, namely the prefrontal cortex, amygdala, and hippocampus. Finally, we consider the behavioral relevance of the longitudinal GMV changes observed in adolescents and focus on traits known to provide risk factors for the occurrence of psychopathology later in life, that is callous unemotional traits and internalizing symptoms^4,33,34^.

## Results

### Effects of early childhood stress

**Figure 2:**
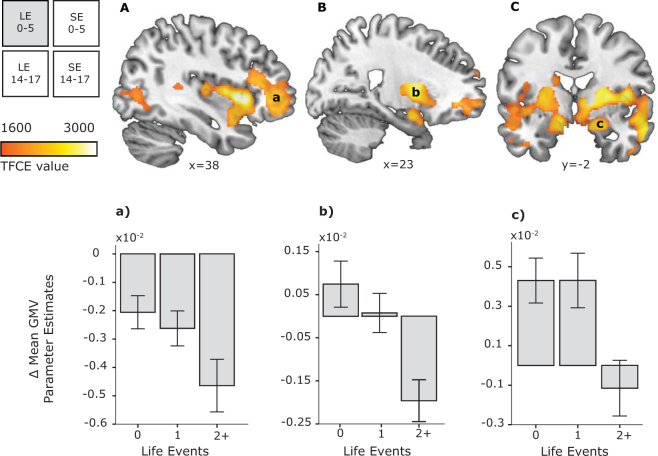
Personal early-life events modulate grey matter volume (GMV) changes in the (**A**) prefrontal cortex, (**B**) insula, and (**C**) amygdala (statistical maps thresholded at TFCE P_FWE_ < 0.05 overlaid on representative structural images). For visualization purposes, the number of adverse life events (LE) was split into three categories: 0, 1, 2+ (two or more) negative events. Graphs show parameter estimates of GMV change between ages 14 and 17. SE, social environment; TFCE, threshold-free cluster-enhancement; FWE, family-wise error; x and y indicate medio-lateral and antero-posterior location of the structural section in stereotactic space, respectively. Error bars represent +/− 1 SE.

Changes in brain structure between ages 14 and 17 were significantly modulated by early-life events. Namely, adolescents who experienced more negative personal early-life events (before age 5) showed larger GMV decreases. These decreases occurred in subcortical structures such as the putamen, insula, caudate, and thalamus, as well as cortical areas spanning the prefrontal, frontal, posterior cingulate and temporal cortex (Fig. 2, Table 1). A similar direction, but different type of GMV changes occurred in the amygdala – more negative personal early-life events were associated with a lack of growth in this region between ages 14 and 17. Having experienced no and one negative personal life event was even associated with an increase in amygdala volume. These effects were not related to baseline differences at age 14 in respect to early-life events (see S2 in Supplementary Information). Variations in early social environment were not associated with significant GMV changes. (See S3 in Supplementary Information for model controlling scanner-type effects.) General developmental changes are addressed in Supplementary Information, Tables S3 and S4.

**Table 1:** Effects of Personal Early-Life Events on grey matter volume changes between age 14 and 17.

Anatomical Region	Side	BA	K	x	y	z	P_FWE_	TFCE	Mean parameter estimates per category		
									0	1	+2
Middle frontal gyrus	L	47/46	5490	−26	46	9	0.022	2044	−0.0021	−0.0027	−0.0049
Frontal pole	L	11		−20	62	−9	0.029	1852	−0.0014	−0.0021	−0.0060
Superior frontal gyrus	L	11		−18	51	3	0.029	1837	−0.0006	−0.0012	−0.0017
Posterior cingulate cortex	R	23	1273	3	−36	28	0.034	1746	−0.0033	−0.0042	−0.0071
	L	23		−8	−30	30	0.034	1722	−0.0013	−0.0015	−0.0023
Anterior insula	R	48	29243	38	14	0	0.005	3006	0.0017	−0.0005	−0.0030
Putamen	R			26	2	8	0.008	2751	0.0008	0.0003	−0.0021
Insula	R	48		44	0	8	0.008	2739	−0.0006	−0.0018	−0.0036
Orbitofrontal cortex	R	47		44	45	−15	0.012	2459	−0.0007	−0.0017	−0.0055
Amygdalae	R	34		22	−2	−18	0.012	2429	0.0051	0.0047	−0.0009
	L	34/25		−12	3	−14	0.012	2429	0.0024	−0.0011	−0.0031
Medial parietal cortex	L	7	1260	−8	−64	44	0.040	1648	−0.0016	−0.0032	−0.0049
Postcentral sulcus	L	2		−24	−39	40	0.041	1632	−0.0003	−0.0004	−0.0007
Medial parietal cortex	R	7		3	−68	39	0.042	1625	−0.0029	−0.0041	−0.0058
Supramarginal gyrus	R	48	42	50	−39	32	0.049	1560	−0.0019	−0.0023	−0.0039
Superior temporal gyrus	R	42	5	57	−33	20	0.050	1551	−0.0011	−0.0003	−0.0026
Middle temporal gyrus	L	37	260	−45	−58	9	0.044	1607	−0.0018	−0.0030	−0.0047
Middle occipital gyrus	L	39		−33	−70	15	0.048	1570	−0.0011	−0.0009	−0.0019
Inferior temporal gyrus	R	20	14	56	−14	−34	0.047	1564	−0.0036	−0.0016	−0.0053
Middle occipital gyrus	R	19	2111	39	−72	0	0.032	1797	−0.0004	−0.0014	−0.0025
Inferior temporal gyrus	R	37		52	−66	−6	0.037	1703	−0.0032	−0.0036	−0.0062
Middle temporal gyrus	R	21		50	−46	12	0.037	1699	−0.0037	−0.0043	−0.0056
Inferior occipital gyrus	L	19	317	−34	−74	−8	0.033	1749	−0.0005	−0.0016	−0.0023

BA, Brodmann Area; K, number of voxels in a cluster; P_FWE_, combined peak-cluster level value; TFCE, threshold free cluster enhancement statistic; R, right; L, left. Note:Mean parameter estimates are split into categories of 0, 1, 2 or more early-life events for interpretational purposes. Table presents MNI coordinates of anatomically relevant markers of the cluster. Clusters have more than one local maxima, for a complete list see Supplementary Information Table S1.

### Effects of current adolescent stress

**Figure 3:**
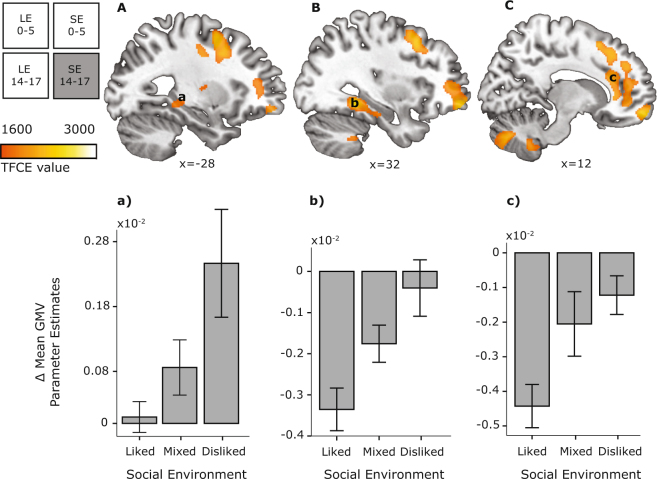
Adolescent peer social environment modulates GMV changes in the (**A**) left hippocampus, (**B**) right parahippocampal gyrus, and (**C**) anterior cingulate cortex (TFCE P_FWE_ < 0.05). For visualization purposes, adolescent peer environment was grouped into three categories: liked (>0.5 on social preference scale), mixed (0 to 0.5 on social preference scale), and disliked (<0 on social preference scale). Graphs show parameter estimates of GMV change between ages 14 and 17. Other conventions as in Fig. 2. Error bars represent +/− 1 SE.

Negative personal life events during adolescence were not associated with significant modulations of grey matter maturation. However, variations in the peer environment were associated with GMV changes in the anterior cingulate, parahippocampus, and prefrontal cortex (Fig. 3, Table 2). Namely, adolescents disliked by their peers showed smaller GMV decreases in those cortical regions, and even an increased GMV in the hippocampus. (See S3 in Supplementary Information for model controlling scanner-type effects.) General developmental changes are addressed in Supplementary Information, Tables S3 and S4.

**Table 2:** Effects of Peer Environment on grey matter volume changes between age 14 and 17.

Anatomical Region	Side	BA	K	x	y	z	P_FWE_	TFCE	Mean parameter estimates per category		
									Liked	Mixed	Disliked
Orbitofrontal cortex	R	11	14197	12	60	−14	0.014	2350	−0.0052	−0.0022	−0.00003
Middle frontal gyrus	R	9		39	10	50	0.016	2290	−0.0041	−0.0035	−0.0004
Frontal pole	R	11		28	56	−12	0.016	2283	−0.0057	−0.0018	0.0004
Anterior cingulate cortex	R	32		9	32	21	0.022	2024	−0.0036	−0.0023	−0.0003
Middle frontal gyrus	L	47	427	−24	39	6	0.030	1845	−0.0002	−0.0001	−0.00007
	L	46		−20	40	15	0.035	1760	−0.001	−0.0007	−0.0004
Inferior frontal gyrus/Middle frontal gyrus	R	46	61	40	34	26	0.045	1648	−0.0053	−0.0034	−0.0019
Fusiform gyrus	R	37	631	32	−42	−9	0.013	2446	−0.0056	−0.0027	−0.0007
Parahippocampal gyrus	R	20		32	−22	−21	0.039	1727	−0.0009	0.001	0.0032
Putamen	L		26	−28	−10	8	0.048	1612	−0.0008	0.00005	−0.0001
Parahippocampal gyrus	L	27	450	−14	−33	−9	0.024	1976	−0.0011	0.0004	0.0020
Hippocampus/Parahippocampal gyrus	L	37		−26	−36	−8	0.035	1772	−0.0001	0.0006	0.0026
Middle temporal gyrus	L	37	237	−58	−66	8	0.028	1900	−0.0046	−0.002	−0.001
Middle temporal gyrus	L	21	305	−52	−44	0	0.031	1840	−0.0063	−0.0038	−0.002
Medulla	L		1093	−2	−48	−63	0.029	1882	0.00003	0.001	0.0019
Cerebellum	R			10	−52	−46	0.029	1877	−0.0012	0.0005	0.0023
Vermis	L/R		1603	0	−63	−34	0.017	2236	−0.0012	0.0012	0.0025
Cerebellum	R			9	−74	−36	0.021	2087	−0.0007	0.00003	0.0026
Cerebellum	L		631	−14	−84	−34	0.032	1817	−0.0002	0.0008	0.0029
Cerebellum	L		12	−9	−50	−46	0.049	1597	−0.0008	0.0002	0.0013
Cerebellum	R		8	20	−22	−30	0.049	1598	0.00008	0.0019	0.0026

BA, Brodmann Area; K, number of voxels in a cluster; P_FWE_, combined peak-cluster level value; TFCE, threshold free cluster enhancement statistic; R, right; L, left. Note:Mean parameter estimates are split into liked (>0.5 on social preference scale), mixed (0 to 0.5 on social preference scale), and disliked (<0 on social preference scale) for interpretational purposes. Table presents MNI coordinates of anatomically relevant markers of the cluster. Clusters have more than one local maxima, for a complete list see Supplementary Information Table S2.

### Interaction effects of early and current stressors

Two additional models tested for the interaction of early and current stressors as well as the effect of socioeconomic status (SES) on GMV changes described above. In the first model, the interaction of negative personal early-life events and the adolescent peer environment did not significantly modulate GMV, showing that early and current stress are independently related to neurodevelopmental maturation. In the second model, SES also did not significantly modulate GMV changes. All effects of early and adolescent stressors described previously remained the same in both models.

### Behavioral relevance of longitudinal GMV changes in adolescents

**Figure 4:**
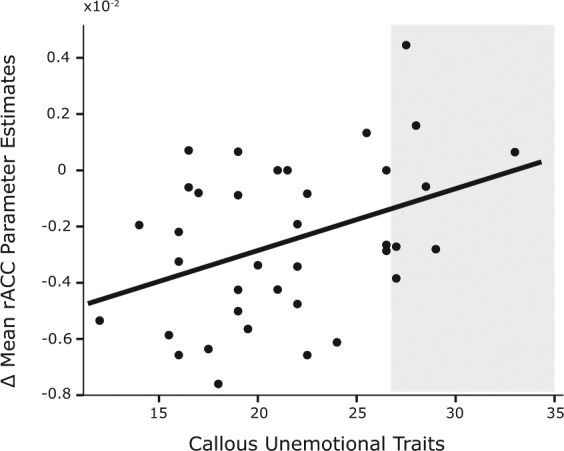
Scatterplot of the association between parameter estimates of GMV change in right anterior cingulate cortex and callous unemotional traits (*r* = 0.39). Each dot represents a participant (*n* = 37). Shaded area represents scores above the cutoff point for individuals at risk^88^.

We also assessed the behavioral relevance of longitudinal GMV changes observed in our adolescent sample. We explored whether volumetric changes in regions affected by early-life stress were related to the presence of internalizing symptoms and adolescent social stress to callous-unemotional traits. Correlational analysis between grey matter volume changes (controlled for early and current life events, early social environment, and gender) and callous-unemotional traits showed a positive relationship localized to the right anterior cingulate cortex (*r* = 0.39, *p* = 0.018, Bayes Factor = 6.05). Namely, a smaller developmental GMV decrease in the right anterior cingulate cortex was associated with the presence of more callous-unemotional traits (Fig. 4). The correlation between right anterior cingulate cortex GMV changes and callous-unemotional traits remained significant when controlling for adolescent social environment (*r* = 0.33, *p* = 0.048). This relationship was not present for the bilateral orbitofrontal cortex (*r* = 0.28, *p* = 0.091, Bayes Factor = 1.53). There were no significant associations between internalizing symptoms and developmental GMV changes (*p* > 0.09).

## Discussion

In this study, we tested the contribution of early childhood and current stress factors on brain maturation of adolescents between mid and late puberty, while differentiating between personal and social stress. Cerebral developmental trajectories were accelerated by early childhood personal stress, and delayed or disrupted by current social stress. Namely, the maturational decrease in GMV over the anterior prefrontal cortex, amygdala, putamen and insula was stronger in those adolescents that experienced negative personal life events during early childhood. In contrast, the maturational decrease in GMV over the anterior cingulate cortex, the parahippocampal gyrus, and the prefrontal cortex was smaller in those adolescents that experience ongoing social stress. Early and current stress were independently related to GMV changes and did not have a cumulative effect on neurodevelopmental maturational profiles. Furthermore, stress-related modulations of the developmental trajectory of the anterior cingulate cortex were already behaviorally relevant, accounting for a significant portion of variance in the adolescents’ antisocial traits.

These observations provide the first empirical evidence that, in typically developing children, even moderate early-life stress can incubate long-lasting effects, leading to increased neural pruning during puberty. The effects of early-life stress are functionally and spatially distinct from current social stress, which modulates neurodevelopmental trajectories in the opposite direction, and over different neural structures. This study also suggests that the neurodevelopmental effects of ongoing social stress may be related to adolescents’ behavioral traits. These findings qualify how pubertal neural plasticity depends on a combination of type and timing of the stressors experienced by a child.

This study shows that negative personal early-life events are associated with larger reductions in subcortical and prefrontal GMV, in line with findings from sub-clinical cohorts of adolescents dealing with severe traumatic events^19,21,35,36^. Elaborating on previous evidence indicating how childhood maltreatment flattens the growth of the amygdala between early to mid-adolescence^10^, here we show that the trajectory of amygdala development continues to be impacted by early-life stress during the second half of puberty. Namely, even moderate negative personal events occurring early in life, such as illness, bias pubertal neurodevelopmental patterns. This bias consists of an increased reduction of grey matter volume, within a prefrontal-amygdala circuit known to control emotional reactivity^6,37^. The direction and location of these findings fit with the notion that early-life stress leads to faster pubertal brain maturation^23,38^, possibly as a consequence of accelerated synaptic pruning^1,39^. Rodent models have shown that early-life stressors alter the regulation of glucocorticoids and hypothalamic corticotropin-releasing factor (CRF), leading to long-term hypothalamic-pituitary-adrenal axis disturbances^40^. Early-life stress may also prematurely activate structures of the emotion regulation circuit, fixating the brain into an adult-like configuration^41^ with precocious myelination of amygdala axons^42^ and earlier emergence of adult-like long term potentiation (LTP)^43^.

Early maturation may be the outcome of an adaptive mechanism at a time of heightened stress^23^. However, it might also prevent the brain from adjusting to the current environment by means of the developmental plasticity usually afforded by adolescence, leading to later costs for mental and physical health^44^. Dendritic spine density in the prefrontal cortex is strongly influenced by stress-related modulations of the noradrenergic system, and of the GABAergic system within the basolateral amygdala and the hippocampus^45,46^. In mice, deviations in dendritic spine density induced during puberty are detrimental for optimal cognition in adulthood^47,48^. The present findings fit with those neurobiological observations and open the way to test whether the GMV changes reported here are driven by structural neuronal changes^49,50^.

In adults, acute stress decreases GMV^25,51,52^. In adolescents, the effects of recent stress on GMV are less clear. While animal models report GMV decreases in the frontal cortex and hippocampus^16^, the handful of existing human studies point to GMV increases in these same regions in children and adolescents suffering from PTSD^53,54^. GMV increases have also been reported in the anterior cingulate cortex, parahippocampal gyrus, and temporal cortex following recent or perceived stress in adolescents and young adults^55,56^; as well as inferior temporal gyrus increases related to social rejection sensitivity in the latter group^57^. Here we add to those findings by showing that, in adolescents, negative peer environment leads to both increased hippocampal GMV as well as a lack of GMV reduction in anterior cingulate cortex and prefrontal cortex. These observations suggest that stress during adolescence delays or disrupts the physiological reduction of GMV previously reported in cortical structures^1,58^.

This study suggests that the effects of current social stress on cingulate development might already influence the emergence of adolescents’ antisocial traits. This observation confirms, on a longitudinal scale, previous cross-sectional studies in children and adolescents reporting an increase in prefrontal GMV in relation to conduct problems and callous unemotional traits^59,60^. A meta-analysis in adults similarly identified increased cingulate gyrus volume as a neural correlate of antisocial behavior^61^. The anterior cingulate gyrus is involved in the control of cognitive and emotional behavior and through its links to the amygdala, involved in affective processing and empathy^62,63^. The delayed or disrupted structural maturation of this region may partially explain the deficiencies in social behavior, especially empathy, observed in those participants with callous unemotional traits. This in turn may be related to the risk of developing psychopathy later in life^33,64^.

This study benefits from the strengths of a longitudinal design allowing for reliable and accurate tests of developmental changes. Our findings indicate that experiencing mildly stressful events early in life can already change neural maturation in puberty. In turn, these findings may help to characterize the developmental processes evoked by traumatic experiences and related emotional problems. However, there are some limitations that should be considered. It might be argued that the reliability of those inferences is limited by the moderate sample size of this study (*n* = 37). However, that limitation on sensitivity should be weighed against the specificity afforded by the accurate characterization of the developmental profile of each participant from birth until 17 years, including neuro-developmental trajectories during puberty.

The findings of this study relate increased GMV reductions to the exposure of early-life stress. This is in contrast to a few adolescent cross-sectional or between-group studies reporting amygdalar or hippocampal GMV increases or null effects^9,54,65,66^. Inconsistencies in the field may be related to stressor-type or measurement period^27^. For example, it has been suggested that early enlargement of the amygdala may occur in response to adversity, later followed by premature volume reduction^26^. We also did not find associations between early-life induced GMV changes and internalizing symptoms while other studies have related structural changes in the prefrontal cortex, ACC and amygdala to internalizing problems^4,67,68^. This may be related to the fact that in this study, we assess developmental trajectories in contrast to generally reported end-point group difference measures. Finally, since increased hippocampal volume related to negative peer environment was particularly affected by measurement on different scanners in our sample, it needs to be replicated in future longitudinal studies and treated tentatively. The longitudinal design of this study, coupled with its focus on healthy children, distinguishes genuine developmental effects from incidental cohort differences. Future follow-up studies might be able to address why some adolescents develop stress susceptibility while others become stress resilient^69^.

## Conclusions

These findings suggest that brain maturation between mid and late adolescence is particularly sensitive to adverse personal events early in life and to adverse social events during adolescence. Increased grey matter reduction in the prefrontal cortex and several subcortical regions was associated with negative personal early-life events. This observation is consistent with the idea that early-life stress accelerates pubertal development. In contrast, brain maturation was disrupted by the effect of concurrent adolescent social stress. This suggests that both early as well as later stressors can bias neurodevelopmental trajectories, which in turn may affect mental health outcomes. Having defined the relative contribution of time-delineated stressors on the maturation of neural circuits during adolescence, this study opens the way to understanding stress susceptibility and resilience later in adulthood.

## Materials and Methods

### Participants

All actively participating children from the Nijmegen Longitudinal Study on Child and Infant Development (*n* = 116) were approached to take part in this imaging study. Anatomical scans were obtained from participants at 14 (*M* = 14.6, *SD* = 0.17) and 17 years of age (*M* = 17.09, *SD* = 0.15). Forty-nine at the first imaging time-point and ninety-six at the second imaging time-point agreed to participate. Participants who could not undergo magnetic resonance imaging (MRI) or who had missing data at one of the two time-points were excluded from these analyses. The final sample consisted of 37 adolescents (15 boys). Participants did not have a history of psychiatric disorders or neurological illness (as indicated by parent/guardian report). Table 3 presents the characteristics of the sample. Written informed consent was obtained from parents and participants during each measurement wave. The study was approved by the local ethics committee (CMO region Arnhem – Nijmegen) and was conducted in compliance with these guidelines.

**Table 3:** Sample characteristics.

	Age (years)	Mean (SD)	Min/Max
***Pubertal Development***			
Testosterone levels [pg/ml]	14	Boys: 42.67 (34.23); Girls: 11.08 (6.52)	4.4/149.4; 1.7/26.54
	17	Boys: 146.43 (73.99); Girls: 23.93 (12.27)	54.06/296.69; 9.10/51.11
PDS	14	Boys: 2.38 (0.33) Girls: 2.89 (0.38)^^^	2/3.2; 2/3.4
	17	Boys: 3.57 (0.38); Girls: 3.40 (0.41)	2.75/4; 2.5/4
***Cognitive Functioning***			
Bayley cognitive development	1.25	108.73 (14.498)	71/137
Peabody verbal ability	5	111.43 (16.227)	82/136
Academic performance TR	16	4.82 (1.36)^^^	1/7
Learning progress TR	16	4.37 (1.19)^^^^	2/7
Adequate school behavior TR	16	4.84 (1.22)^^^^^	2/6
***SES During Childhood***			
Education mother	1.25	5.24 (1.66)	2/7
Education father	1.25	5.27 (1.68)	2/7
Work mother	1.25	3.24 (2.06)	0/6
Work father	1.25	3.81 (1.49)	0/6
***Measures of Interest***			
Personal early-life events	0–5	1.43 (1.19)	0/4
Parent-child interaction scores	0–5	−0.06 (3.05)	−7.86/4.61
Personal current life events	14–17	1 (1.05)	0/4
Peer ratings	16	0.15 (0.76)	−2.18/1.33
Internalizing symptoms [CBCL]	17	raw scores: 5.51 (5.71); T scores: 50 (9.94)	0/28; 33/75
Callous unemotional traits [ICU]	17	21.46 (4.94)	12/33

PDS, Pubertal Development Scale; SES, social economic status; TR, teacher report, based on a 7-point scale (S1 in Supplementary Information provides details on Cognitive Functioning measures); CBCL, Child Behaviour Checklist; ICU, Inventory of Callous Unemotional Traits. ^^^n = 34; ^^^^n = 30; ^^^^^n = 32. Note: There were no significant correlations (p < 0.05) of SES with any early-life or current stressors, pubertal development (testosterone values at age 14 and 17), nor symptomatology (internalizing symptoms, ICU). The restricted range of SES scores in this sample is fairly representative of the Dutch population of families with children in the same age range (for more information on the wider NLS sample and SES see^75^). Concerning associations between cognitive functioning and stress, childhood IQ scores (Peabody) at age 5 were not significantly correlated with early-life stressors assessed up until this age (amount of negative personal events [r = −0.01, p = 0.953]; parent-child interaction quality [r = 0.165, p = 0.33]). The three indices of cognitive functioning during adolescence were also not correlated with either early or later stressors (p > 0.05). The amount of early-life events was moderately correlated with current events (r = 0.332, p = 0.045).

### Life events

The experience of early-life events (before age 5) and current life events (between age 14 and 17) were assessed via parent report. All life event reports were collected within one to two years after the event had taken place. This meant that for early-life events, reports were taken at 15 months, 28 months, and 5 years. For current adolescent life events, the report was taken at age 17 for reports until age 14. The life events questionnaire consisted of items selected from Sarason, Johnson, and Siegel’s Life Experiences Survey^70^ and Coddington’s Life Events Scale for Children^71^ based on the likelihood they would have an aversive influence on the child’s development^72^. Both measures have been widely used in international research^73,74^. The life events questionnaire remained the same at all measurement times and has previously been used in this longitudinal study^75,76^. Items require a ‘yes’ or ‘no’ response. The score represents the total number of negative personal life events in the given assessment period and was calculated for events that took place until early childhood (until age 5) and during late adolescence (i.e., between ages 14 and 17).

### Social environment

We used age-relevant measures of the individual’s social environment (SE). The quality of parent-child interactions was used as an index of early SE. Poor parent-child interaction quality has previously been shown to be related to elevated childhood cortisol levels in the NLS cohort^29^. Parent-child interactions were assessed at 15 months, 28 months, and 5 years of age during a home visit^29,75^. Video recordings of these interactions were rated by four trained observers on five 7-point scales: Supportive Presence, Respect for Child’s Autonomy, Structure and Limit Setting, Quality Instruction, and Hostility. An average score for SE before age 5 was taken across all scales (with reversed coding for hostility scores) and time-points for each child^77^. In case of a missing assessment (*n* = 2 cases) the average was computed based on the two remaining time points.

Peer environment (social preference) between age 14 and 17 was assessed in the classroom with a well-established sociometric measure previously used in this cohort^78,79^. Children were asked to nominate classmates who they liked (“Who do you like the most?”) and disliked (“Who do you like the least?”). Students were asked to nominate at least one classmate, excluding self-nominations. There was no maximum number of nominations. For each question, the number of nominations that a child received was counted and standardized within the classroom, to control for differences in classroom size. A score for social preference was calculated by subtracting the liked least from the liked most score. This difference score was again standardized within classrooms.

### Socioeconomic Status

SES scores were computed based on education (7-point scale) and occupation (6-point scale) levels for both parents in line with previous reports on this cohort^75^. The levels of education and occupation for the two parents were first standardized and then summed to create a single score per parent. The final SES score was derived by taking the average score of the mother and father.

### Behavioral measures of psychopathology

Internalizing symptoms at age 17 were measured using the Child Behaviour Checklist (CBCL)^80^. The CBCL is a parent-report questionnaire used to assess the frequency of emotional and behavioral problems exhibited by the adolescent in the past six months. The parent rated each behavior or symptom on a three-point Likert scale (not true, somewhat or sometimes true, very true or often true). Items from the scales anxious/depressive, withdrawn/depressive, and somatic complaints were summed to provide a score for internalizing symptoms.

Specific aspects of socialization during adolescence was measured with the Inventory of Callous Unemotional Traits ^81^. Self-report and parent-report versions of the questionnaire were used to assess the occurrence and intensity of affective features of callousness such as lack of empathy, disregard for others, and shallow affect. It consisted of 24 items scored on a four-point Likert scale (not at all true, somewhat true, very true, definitely true). The self-report and parent-report versions were significantly correlated with each other (*r* = 0.42, *p* = 0.013). A mean score was created from both versions to increase consistency of the measure. For two participants with missing data (1 self-report, 1 parent-report) the available score was used for further analysis.

The statistical threshold for correlations of GMV with psychopathology measures were set to p < 0.025 (multiple correction for number of regions tested for each measure).

### Imaging parameters

Structural T1 images were acquired at 3 Tesla using Siemens MAGNETOM Trio or PRISMA systems (acquired at the same site; 18 participants at age 17) with a 32-channel coil. Images were acquired using the same MPRAGE sequence (TR = 2300 ms; TE = 3.03 ms; 192 sagittal slices; 1.0 × 1.0 × 1.0 mm voxels; FOV = 256 mm). To ensure that there were no differences in the quality of T1 images acquired on the TRIO and PRISMA scanners at age 17, these normalized and smoothed GM images were checked using the “Check sample homogeneity” function in CAT12 (Computation Anatomy Toolbox). One participant was identified as a potential outlier for manual inspection. After manually checking the data for artefacts, it was included in the analyses.

### Voxel Based Morphometry

Magnetic resonance images were processed using the Matlab toolbox SPM12 [Statistical Parametric Mapping (www.fil.ion.ucl.uk/spm)]. Each MR image was checked for artifacts or anatomical abnormalities and alignment to the anterior commissure. Using a pairwise longitudinal registration approach^82^ a Jacobian difference map was generated as well as a “halfway space” image, which was subsequently segmented into white matter, grey matter (GM), and cerebrospinal fluid (CSF). Diffeomorphic anatomical registration through exponentiated lie algebra (DARTEL) was used for inter-subject registration of the GM “halfway” images to a group average template image^83^. The GM “halfway” image was multiplied by the Jacobian difference map for each participant. This subsequent GM difference map was transformed and resampled at an isotropic voxel size of 1.5 mm, resulting in spatially normalized, Jacobian scaled, and smoothed (8 mm FWHM Gausian kernel) images in Montreal Neurological Institute (MNI) space. Data quality of normalized and smoothed difference images was checked with CAT12 using the “Check sample homogeneity” function. This function did not indicate any potential outliers - based on a mean correlation of the sample below 2 standard deviations. The GM images were entered into a multiple regression analysis with standardized scores of life events and social environment as early (0–5 years) and current (14–17 years) stressors entered as covariates. Gender and average (age 14 and 17) grey and white matter total brain volume (TBV) were entered as covariates of no interest. To minimize boundary effects, a binary mask of the group template was used to exclude voxels outside of the brain. Statistical significance was assessed using non-parametric permutation tests using the Threshold Free Cluster Enhancement (TFCE) Toolbox in SPM12 (Version 90; http://dbm.neuro.uni-jena.de/tfce/) with 5000 permutations. After TFCE, the statistical threshold was set to *p* < 0.05 adjusted for family wise error at a whole brain level. TFCE suppresses random noise that may have a similar intensity as the real signal, but lacks spatial continuity (smoothness). The TFCE values at each voxel represent a combination of spatially distributed cluster size and height information. In other words, the TFCE statistic summarizes the cluster-wise evidence at each voxel. There is no initial threshold for voxel level inference. Statistical inference is based on the distribution of TFCE values - derived from the non-parametric permutations. This type of approach is particularly beneficial for VBM data^84,85^. Anatomical inference was drawn by superimposing images on a standard SPM single-subject T1 template, the group-specific average template (created in DARTEL), and subject-specific T1 scans standardized in MNI space.

### Behavioral relevance of longitudinal GMV changes

To relate the longitudinal GMV changes observed in adolescents to psychopathology, that is callous unemotional traits and internalizing symptoms, GMV changes were extracted from the relevant significant clusters. To achieve anatomical specificity, an overlap was taken between the significant cluster and the brain area, based on the Automated Anatomical Labeling (AAL) Atlas^86^. As such, the grey matter estimates reflected only the significant changes in an anatomically defined area. To test the association between adolescent-social-stress-related volumetric changes and callous unemotional traits, we identified regions significantly modulated by adolescent social stress that have previously been also identified as part of the callous unemotional neuro-profile in adolescent samples^59,60,87^, namely the anterior cingulate cortex (only the right hemisphere in our study) and bilateral orbital frontal cortex. Parameter estimates of grey matter volume changes of these two regions were entered into SPSS and JASP for correlational analysis.

To test the association between early-life stress-induced volumetric changes and internalizing symptomology, we identified regions modulated by negative personal early-life events that have previously been suggested as developmental targets for internalizing disorders, namely the amygdala-prefrontal circuit and anterior cingulate cortex^4,67,68^. GMV changes were extracted from these relevant clusters and analyzed for associations with internalizing symptoms, following the same procedure described for callous unemotional traits.

Finally, two post-hoc analyses were conducted to rule out the effects of additional stressors. The first model included standardized SES scores as an additional regressor in the previously described multiple regression analysis. The second model explored the interaction between early and current stressors. The standardized interaction scores of personal early-life events and adolescent peer environment (the two significant predictors of GMV changes) were entered into the original multiple regression analysis. For both analyses, all other parameters were kept the same as in the original model.

### Data Availability

The data that support the findings of this study are available from the corresponding author upon request.

### Code Availability

The code used to analyze the data is available from the corresponding author upon request.

## Electronic supplementary material

Supplementary Information
