# Supplementary material for: Early-life and pubertal stress differentially modulate grey matter development in human adolescents
Source: Sci Rep. 2018 Jun 15;8:9201. doi: 10.1038/s41598-018-27439-5 (PMC6003940; doi:10.1038/s41598-018-27439-5)
Supplement: Supplementary file 1 — Supplementary Information [file 41598_2018_27439_MOESM1_ESM.pdf]

Manuscript Title: Early-life and pubertal stress differentially modulate grey matter development in human adolescents

Authors: Anna Tyborowska[a,b*], Inge Volman[c], Hannah C. M. Niermann[a,b], J. Loes Pouwels[a], Sanny Smeekens[a,d], Antonius H. N. Cillessen[a], Ivan Toni[b^], Karin Roelofs[a,b^]

Table S1. Effects of **Personal Early-Life Events** on grey matter volume changes between ages 14 and 17.

**Anatomical Region**	**Side**	**BA**	**k**	**x**	**y**	**z**	P_{FWE}	**TFCE**	**Mean parameter estimates per category**		
									**0**	**1**	**+2**
Anterior Insula	R	48	29243	38	14	0	.005	3006	0.0017	-0.0005	-0.0030
		48		39	18	-2	.005	2979	0.0005	-0.0009	-0.0048
Putamen	R			26	2	8	.008	2751	0.0008	0.0003	-0.0021
				21	3	9	.008	2749	0.0008	0.0004	-0.0012
Insula	R	48		44	0	8	.008	2739	-0.0006	-0.0018	-0.0036
Putamen	R			16	12	0	.009	2646	0.0010	$0.4x10^{-4}$	-0.0024
Thalamus/Pallidum	R			15	-2	2	.009	2644	$0.9x10^{-4}$	$0.9x10^{-4}$	$-0.9x10^{-4}$
Middle temporal gyrus	R	21		57	3	-18	.009	2621	-0.0012	-0.0024	-0.0053

		21	62	-4	-22	.011	2501	-0.0020	-0.0024	-0.0051
Orbitofrontal cortex	R	47	44	45	-15	.012	2459	-0.0007	-0.0017	-0.0055
Parahippocampal Gyrus	L	28/34	-12	-2	-16	.012	2435	0.0018	0.0007	-0.0018
Putamen	L		-26	-10	8	.012	2433	0.0001	-0.3×10^{-4}	-0.0004
Thalamus	L		-21	-12	6	.012	2431	0.4×10^{-4}	0.6×10^{-5}	-0.0001
Amygdalae	R	34	22	-2	-18	.012	2429	0.0051	0.0047	-0.0009
	L	34/25	-12	3	-14	.012	2429	0.0024	-0.0011	-0.0031
Thalamus	R		9	-3	0	.013	2397	0.0008	0.0006	-0.0002
Putamen	L		-22	-8	15	.013	2376	0.2×10^{-4}	-0.2×10^{-4}	-0.0002
			-26	2	4	.013	2371	0.0016	-0.0007	-0.0032
Caudate	L		-8	15	-4	.013	2368	-0.0001	-0.001	-0.0063
Amygdala	R	34	27	6	-20	.013	2357	0.0047	0.0044	-0.0035
Olfactory cortex	L	25	-2	6	-12	.014	2346	0.0023	-0.0002	-0.0023
Temporal pole	R	38	48	9	-20	.015	2338	-0.0007	-0.0013	-0.0028
Anterior insula	R	48	33	10	-14	.015	2336	0.0043	0.0019	-0.0010
Temporal pole	R	38	44	10	-18	.015	2327	-0.0002	-0.0009	-0.0028
		38	34	10	-20	.016	2305	0.0029	0.0004	-0.0037

Inferior frontal gyrus	R	48/45		51	20	9	.016	2297	-0.0021	-0.0015	-0.0036
Olfactory	R	38		2	14	-6	.016	2295	0.6x10^{-4}	-0.0011	-0.0037
Frontal pole	R	11		28	54	-10	.016	2266	-0.2x10^{-4}	-0.0017	-0.0040
Rolandic operculum	R	48		57	6	8	.016	2263	-0.0006	-0.0011	-0.0028
Frontal pole	R	11		27	48	-8	.016	2259	-0.0001	-0.0009	-0.0018
Inferior frontal gyrus	R	47		42	46	0	.016	2258	-0.0038	-0.0035	-0.0065
Orbitofrontal cortex	R	47/11		24	42	-4	.016	2257	-0.6x10^{-4}	-0.0002	-0.0004
Middle frontal gyrus	L	47/46	5490	-26	46	9	.022	2044	-0.0021	-0.0027	-0.0049
Frontal pole	L	11		-20	62	-9	.029	1852	-0.0014	-0.0021	-0.0060
Superior frontal gyrus	L	11		-18	51	3	.029	1837	-0.0006	-0.0012	-0.0017
Anterior cingulate cortex	L/R	10		0	48	3	.031	1813	-0.0017	-0.0022	-0.0046
	R	24		3	39	15	.031	1809	-0.0002	-0.0018	-0.0040
Orbitofrontal cortex	L	47		-46	36	-14	.031	1802	-0.0015	-0.0030	-0.0058
Anterior cingulate cortex	L	10		-9	50	2	.032	1781	-0.0021	-0.0036	-0.0051
Medial superior frontal gyrus	L	10		-10	63	8	.032	1779	-0.0006	-0.0016	-0.0039
Inferior frontal gyrus	L	47		-40	34	2	.032	1778	-0.0015	-0.0018	-0.0027
	L	45		-40	28	12	.032	1776	-0.0031	-0.0032	-0.0047

Medial superior frontal gyrus	R	10	2	62	6	.032	1775	-0.0018	-0.0030	-0.0040
	R	10	3	57	6	.032	1774	-0.0015	-0.0035	-0.0046
	R	10	8	63	15	.032	1771	-0.0010	-0.0029	-0.0055
Superior frontal gyrus	R	10	15	64	18	.032	1770	0.0006	-0.0003	$-0.9x10^{-4}$
Medial superior frontal gyrus	L	10	-2	64	18	.032	1767	-0.0018	-0.0026	-0.0036
	R	10	4	62	10	.032	1767	-0.0013	-0.0030	-0.0048
	R	10	3	66	10	.032	1767	-0.0012	-0.0023	-0.0038
Inferior frontal gyrus	L	45	-54	28	15	.033	1758	-0.0023	-0.0023	-0.0050
Anterior cingulate cortex	R	24	2	32	15	.033	1754	0.0011	$0.6x10^{-4}$	-0.0021
Inferior frontal gyrus	L	45	-48	28	15	.033	1750	-0.0042	-0.0032	-0.0062
Middle frontal gyrus	L	10/46	-28	57	14	.034	1748	-0.0043	-0.0051	-0.0067
Inferior frontal gyrus	L	45	-56	27	10	.035	1736	-0.0024	-0.0017	-0.0048
Orbitofrontal cortex	L	45/47	-45	40	-2	.036	1716	-0.0026	-0.0042	-0.0060
Medial orbitofrontal cortex	R	11	4	40	-9	.037	1708	-0.0010	-0.0041	-0.0047
	R	11	0	44	-9	.037	1706	-0.0017	-0.0054	-0.0056
Superior frontal gyrus	L	10	-14	66	15	.038	1684	-0.0021	-0.0029	-0.0052
	R	10/46	24	62	22	.039	1662	-0.0021	-0.0045	-0.0051

	L	10		-18	62	22	.043	1616	-0.0032	-0.0038	-0.0047
	R	10		27	62	18	.045	1591	-0.0023	-0.0048	-0.0055
Medial orbitofrontal gyrus	R	11		2	64	-3	.048	1568	-0.0016	-0.0027	-0.0033
Middle occipital gyrus	R	19	2111	39	-72	0	.032	1797	-0.0004	-0.0014	-0.0025
	R	19		32	-70	0	.032	1781	$-0.8x10^{-4}$	-0.0002	-0.0004
Inferior temporal gyrus	R	19/37		44	-69	-4	.036	1725	-0.0018	-0.0031	-0.0045
	R	37		52	-66	-6	.037	1703	-0.0032	-0.0036	-0.0062
Middle temporal gyrus	R	21		50	-46	12	.037	1699	-0.0038	-0.0043	-0.0056
Fusiform gyrus	R	19/37		32	-64	-4	.038	1680	-0.0004	-0.0009	-0.0011
Middle temporal gyrus	R	37		54	-57	6	.040	1651	-0.0030	-0.0033	-0.0058
Middle occipital gyrus	R	18/19		36	-84	4	.040	1646	-0.0020	-0.0026	-0.0032
Superior temporal gyrus	R	41		40	-36	10	.041	1632	-0.0012	-0.0015	-0.0024
	R	41		42	-40	8	.042	1625	-0.0027	-0.0036	-0.0048
Middle temporal gyrus	R	37		45	-58	8	.045	1591	-0.0031	-0.0029	-0.0049
Inferior occipital gyrus	L	19	317	-34	-74	-8	.033	1749	-0.0005	-0.0016	-0.0023
Post cingulate cortex	R	23	1273	3	-36	28	.034	1746	-0.0033	-0.0042	-0.0071
	L	23		-8	-30	30	.034	1722	-0.0013	-0.0015	-0.0023

	R	26		10	-42	22	.038	1687	-0.0003	-0.0006	-0.0016
Medial parietal cortex	R	26/29		8	-44	20	.038	1686	-0.0012	-0.0016	-0.0032
Post cingulate cortex	R	23/26		9	-40	27	.038	1684	-0.0013	-0.0020	-0.0033
Calcarine gyrus	L	17		-10	-60	9	.044	1610	-0.0027	-0.0033	-0.0040
Medial parietal cortex	L	30		-2	-56	16	.045	1590	-0.0043	-0.0058	-0.0069
Middle cingulate cortex	R/L	23		0	-20	36	.046	1581	-0.0056	-0.0052	-0.0068
Medial parietal cortex	L	7	1260	-8	-64	44	.040	1648	-0.0016	-0.0032	-0.0049
	L	7		-14	-63	42	.041	1635	-0.0005	-0.0016	-0.0022
Postcentral sulcus	L	2/7/40		-24	-39	40	.041	1632	-0.0003	-0.0004	-0.0007
Postcentral sulcus	L	2/7/40		-26	-39	36	.042	1629	-0.0002	-0.0003	-0.0006
Medial parietal cortex	R	7		3	-68	39	.042	1625	-0.0029	-0.0041	-0.0058
	L	23		-9	-57	27	.043	1616	-0.0028	-0.0034	-0.0049
Intraparietal sulcus	L	7/40		-26	-45	32	.044	1612	-0.0001	-0.0002	-0.0004
	L	7/40		-21	-52	36	.044	1611	-0.0003	-0.0005	-0.0006
	L	7/40		-26	-50	32	.044	1608	-0.0002	-0.0003	-0.0004
Medial parietal cortex	L	7		-9	-68	34	.044	1600	-0.0024	-0.0035	-0.0042
Cuneus	L	7/19		-12	-75	36	.045	1597	-0.0015	-0.0021	-0.0044

Postcentral gyrus	L	40		-36	-35	46	.046	1580	-0.0040	-0.0038	-0.0060
Cuneus	L	18/19		-9	-80	30	.048	1571	0.0002	-0.0007	-0.0021
Middle temporal gyrus	L	37	260	-45	-58	9	.044	1607	-0.0018	-0.0030	-0.0047
Middle occipital gyrus	L	39		-33	-70	15	.048	1570	-0.0011	-0.0009	-0.0019
Middle occipital gyrus	L	19/37		-38	-74	12	.048	1567	-0.0018	-0.0017	-0.0031
Inferior temporal gyrus	R	20	14	56	-14	-34	.047	1574	-0.0036	-0.0016	-0.0053
Supramarginal gyrus	R	48	42	50	-39	32	.049	1560	-0.0019	-0.0023	-0.0039
Superior temporal gyrus	R	42	5	57	-33	20	.050	1551	-0.0011	-0.0003	-0.0026

BA, Brodmann Area; k, number of voxels in a cluster; p_{FWE}, combined peak-cluster level value; TFCE, threshold free cluster enhancement statistic; R, right; L, left.

Note: Mean parameter estimates are split into categories of 0, 1, 2 or more early-life events for interpretational purposes. Table presents clusters' local maxima more than 4 mm apart.

Table S2. Effects of **Peer Environment** on grey matter volume changes between ages 14 and 17.

Anatomical Region	Side	BA	k	x	y	z	p_{FWE}	TFCE	Mean parameter estimates per category		
									Liked	Mixed	Disliked
Fusiform gyrus	R	37	631	32	-42	-9	.013	2446	-0.0056	-0.0027	-0.0007
Parahippocampal gyrus	R	20		32	-22	-21	.039	1727	-0.0009	0.0010	0.0032
Orbitofrontal cortex	R	11	14197	12	60	-14	.014	2350	-0.0052	-0.0022	$-0.3x10^{-4}$
Middle frontal gyrus	R	9		39	10	50	.016	2290	-0.0041	-0.0035	-0.0004
Frontal pole	R	11		28	56	-12	.016	2283	-0.0057	-0.0018	0.0005
Middle frontal gyrus	R	9		33	14	45	.017	2267	-0.0026	-0.0016	-0.0003
Precentral gyrus	R	6		48	-2	39	.017	2263	-0.0017	$0.5x10^{-4}$	0.0011
Middle frontal gyrus	L	9		-36	10	45	.017	2238	-0.0046	-0.0024	$-0.6x10^{-4}$
Precentral gyrus	L	6		-30	3	50	.018	2195	-0.0041	-0.0028	-0.0010
Superior frontal gyrus	L	6		-14	14	51	.018	2181	-0.0023	-0.0011	-0.0003
Precentral gyrus	L	6		-44	2	45	.018	2172	-0.0063	-0.0036	-0.0008
Superior frontal gyrus	L	32		-12	34	44	.018	2163	-0.0037	-0.0014	-0.0002
	R	8		20	14	54	.018	2161	-0.0051	-0.0046	-0.0009

Middle frontal gyrus	L	6/44	-27	9	38	.019	2143	-0.0009	-0.0007	-0.0003
Superior frontal gyrus	R	32	15	26	44	.019	2126	-0.0016	-0.0014	-0.0006
	R	32	15	27	38	.020	2110	-0.0013	-0.0009	-0.0004
Inferior frontal gyrus / Middle frontal gyrus	R	44	44	14	38	.020	2102	-0.0056	-0.0028	-0.0019
Precentral gyrus	R	44	46	10	36	.020	2101	-0.0052	-0.0028	-0.0022
Superior frontal gyrus	R	9	21	20	32	.020	2097	-0.0003	-0.0002	-0.9×10^{-4}
	L	32	-12	40	34	.020	2092	-0.0024	-0.0015	-0.0009
	L	6	-18	6	57	.021	2085	-0.0049	-0.0032	-0.0001
	L	6	-22	4	58	.021	2085	-0.0062	-0.0049	-0.0005
Frontal pole	R	47	40	52	-9	.021	2085	-0.0058	-0.0019	-0.0007
Medial superior frontal gyrus	L	9/32	-9	45	33	.021	2076	-0.0032	-0.0016	-0.0012
	L	32	-8	45	26	.021	2072	-0.0048	-0.0033	-0.0015
Supplementary motor area	L	32	-10	22	50	.021	2061	-0.0029	-0.0015	-0.0007
Medial superior frontal gyrus	R	9/32	9	44	34	.021	2052	-0.0037	-0.0016	-0.0006
Precentral gyrus	L	6	-46	-4	39	.022	2033	-0.0025	-0.0006	0.0002
Anterior cingulate cortex	R	32	9	32	21	.022	2024	-0.0036	-0.0023	-0.0003

Rectus gyrus	L	11		-2	57	-15	.022	2024	-0.0046	-0.0031	-0.0020
Anterior cingulate cortex	R	32		15	27	20	.023	2013	-0.0004	-0.0004	0.0001
	R	11/32		12	33	3	.023	2000	-0.0004	-0.8x10^{-4}	0.0003
Medial orbitofrontal cortex	R	10/11		2	63	-8	.023	1998	-0.0039	-0.0026	-0.0018
Anterior cingulate cortex	R	25		8	28	2	.024	1991	-0.0004	-0.0001	0.0006
Vermis	R/L		1603	0	-63	-34	.017	2236	-0.0012	0.0012	0.0025
Cerebellum	R			9	-74	-36	.021	2087	-0.0007	0.3x10^{-4}	0.0026
	R			14	-80	-46	.028	1893	-0.0026	-0.0031	0.0013
Vermis	R			2	-50	-28	.033	1799	0.3x10^{-4}	0.0007	0.0013
Cerebellum	L			-6	-72	-30	.034	1788	-0.0008	0.0014	0.0028
Vermis	R/L			0	-44	-30	.049	1606	0.6x10^{-4}	0.0005	0.0007
Parahippocampal gyrus	L	27	450	-14	-33	-9	.024	1976	-0.0011	0.0004	0.0020
Hippocampus / Parahippocampal gyrus	L	37		-26	-36	-8	.035	1772	-0.1x10^{-3}	0.0006	0.0026
Middle temporal gyrus	R	37	237	-58	-66	8	.028	1900	-0.0046	-0.0021	-0.0011
Medulla	L		1093	-2	-48	-63	.029	1882	0.3x10^{-4}	0.0010	0.0019
Cerebellum	R			10	-52	-46	.029	1877	-0.0012	0.0005	0.0023

	R			3	-52	-54	.029	1868	-0.0015	-0.0006	0.0021
	R			20	-48	-39	.033	1801	$0.3x10^{-4}$	$0.7x10^{-4}$	0.0003
	R			36	-45	-44	.033	1790	-0.0002	-0.0001	0.0025
	R			26	-45	-40	.034	1780	0.0003	0.0004	0.0011
	R			18	-44	-36	.037	1745	$0.4x10^{-4}$	$0.8x10^{-4}$	0.0002
	R			48	-50	-46	.045	1641	-0.0004	$0.6x10^{-4}$	0.0021
	R			21	-39	-28	.049	1601	-0.0008	-0.0007	0.0009
	R			21	-39	-33	.049	1600	-0.0003	-0.0002	0.0003
Middle frontal gyrus	L	47	427	-24	39	6	.030	1845	-0.0002	-0.0001	$-0.7x10^{-4}$
	L	46		-20	40	15	.035	1760	-0.0010	-0.0007	-0.0004
Middle temporal gyrus	L	21	305	-52	-44	0	.031	1840	-0.0063	-0.0038	-0.0021
Cerebellum	L		631	-14	-84	-34	.032	1817	-0.0002	0.0008	0.0029
	L			-9	-81	-42	.032	1803	-0.0019	-0.0016	0.0022
	L			-15	-81	-48	.033	1791	-0.0019	-0.0029	0.0017
	L			-12	-82	-45	.033	1789	-0.0016	-0.0020	0.0027
	L			-15	-93	-32	.042	1690	0.0002	$-0.2x10^{-4}$	0.0015
Inferior frontal gyrus / Middle	R	46	61	40	34	26	.045	1648	-0.0053	-0.0034	-0.0019

frontal gyrus										
Putamen	L	26	-28	-10	8	.048	1612	-0.0008	$0.5x10^{-4}$	-0.0001
Cerebellum	R	8	20	-22	-30	.049	1598	$0.8x10^{-4}$	0.0020	0.0026
Cerebellum	L	12	-9	-50	-46	.049	1597	-0.0008	0.0002	0.0013

BA, Brodmann Area; k, number of voxels in a cluster; p_{FWE}, combined peak-cluster level value; TFCE, threshold free cluster enhancement statistic; R, right; L, left.

Note: Mean parameter estimates are split into liked (>0.5 on social preference scale), mixed (0 to 0.5 on social preference scale), and disliked (< 0 on social preference scale) for interpretational purposes. Table presents clusters' local maxima more than 4 mm apart.

Table S3. Decreases [a] in grey matter volume between age 14 and 17

**Anatomical Region**	**Side**	**BA**	**K**	**x**	**y**	**z**	**P_{FWE}**	**TFCE**
Superior frontal gyrus	L	9	135	-3	48	38	.038	1283
Superior temporal pole	L	38	377	-46	24	-14	.031	1375
Orbital frontal gyrus	L	38/47		-36	18	-16	.046	1192
Inferior temporal gyrus	L	20	2142	-63	-32	-20	.010	1904
Middle temporal gyrus	L	20		-51	-10	-15	.026	1447
	L	20		-51	2	-30	.028	1417
Middle temporal gyrus	L	21	140	-63	-54	15	.041	1251
	L	37		-60	-62	16	.045	1215
	L	37		-58	-66	8	.046	1203

BA, Brodmann Area; k, number of voxels in a cluster; p_{FWE}, combined peak-cluster level value; TFCE, threshold free cluster enhancement statistic; R, right; L, left.

Note: Table presents clusters' local maxima more than 8 mm apart.

[a] Controlled for early and current personal life events, parent-child interactions, peer environment, and gender.

Table S4. Increases [a] in grey matter volume between age 14 and 17

Anatomical Region	Side	BA	K	x	y	z	P_{FWE}	TFCE
Medulla	L/R		200	0	-40	-56	.016	1664
Medulla	R		171	8	-34	-38	.024	1508
Cerebellum	R			2	-40	-38	.033	1369
Pons	R		240	8	-15	-20	.020	1585
	R		32	15	-10	-33	.047	1236
	R		4	9	-14	-30	.040	1304

BA, Brodmann Area; k, number of voxels in a cluster; p_{FWE}, combined peak-cluster level value; TFCE, threshold free cluster enhancement statistic; R, right; L, left.

Note: Table presents clusters' local maxima more than 8 mm apart.

[a] Controlled for early and current life events, early social environment, and gender.

S1. The Dutch version of the Bayley [1] Mental Scale of Infant Development [2] was used to assess the child's level of cognitive functioning at 15 months. Scores are expressed in the standardized Mental Developmental Index (MDI; M = 100; SD = 15). The Peabody Picture Vocabulary Test - Revised [3] was used to measure verbal intelligence at age 5. Like other standard IQ tests, scores are on a scale with a mean of 100 and a standard deviation of 15. Cognitive functioning indices at age 16 were taken from the Teacher Report Form [4] (Dutch version [5]). Answers were scored on a 7-point Likert scale (1 – far below the average, 7 – far above the average). Academic performance is based on the question, "How does he/she perform compared to the rest of the class?". Learning progress is based on the question, "How does he/she progress with learning?". Adequate school behavior is based on the question, "How adequately does he/she behave?"

S2. To ensure that the observed effects of early life stress were not related to baseline differences in GMV, we separately tested whether personal early-life events and parent-child interactions were related to GMV intra-subject differences at age 14. Each MR image was checked for artifacts or anatomical abnormalities and alignment to the anterior commissure. The anatomical images were segmented into grey matter, white matter, and CSF in SPM12. DARTEL [6] was used for inter- subject registration of the grey matter images to a group template. The registered images were smoothed (8mm FWHM Gaussian kernel, Jacobian scaled, threshold at 0.2) and transformed into MNI space. The GM images were entered into a multiple regression analysis with standardized scores of personal early-life events and social environment (parent-child interactions) as covariates. Gender and total brain volume (TBV) were entered as covariates of no interest. To minimize boundary effects, a binary mask of the group template was used to exclude voxels outside of the brain. Following previous analyses in the manuscript, TFCE was used to assess statistical significance, with the threshold set to $p<.05$, family-wise

error corrected at the whole-brain level. We did not find any significant effects in GMV differences for personal early-life events nor for parent-child interaction scores.

S3. An additional multiple regression analysis was conducted to check for scanner effects. Scanner type (TRIO vs PRISMA) was entered as a condition-specific factor for personal early-life events and social preference. The remainder of the model remained the same (parent-child interactions, personal current life events, gender, and average total brain volume entered as covariates). The effects of personal early-life events remained the same as described in the manuscript. Social preference results remained similar, but with reduced cluster size. As a result, the right anterior cingulate cortex cluster was present at a subthreshold level (p_{FWE} = .057). The left hippocampus cluster was no longer present after scanner correction. This finding should therefore be treated tentatively and needs to be replicated in future studies.

References

1. Bayley, N. *Manual for the Bayley Scales of Infant Development*. (Psychological Corporation, 1969).

2. van der Meulen, B. F. & Smrkovsky, M. *Bayley Ontwikkelingsschalen: Handleiding*. (Swets & Zeitlinger, 1983).

3. Dunn, L. M. & Dunn, L. M. *Peabody Picture Vocabulary Test, revised: Forms L and M*. (American Guidance Service, 1981).

4. Achenbach, T. M. & Rescorla, L. A. *Manual for the ASEBA*. (University of Vermont, Research Center for Children, Youth, and Families, 2001).

5. Verhulst, C., Frank, C., van der Ende, J. & Koolhans, M. *Teacher Report Form voor kinderen 6-18 jaar*. (Sophia Kinderziekenhuis, 2001).

6. Ashburner, J. A fast diffeomorphic image registration algorithm. *Neuroimage* **38,** 95–113 (2007).
